# An Intra-Individual Comparison of Low-keV Photon-Counting CT versus Energy-Integrating-Detector CT Angiography of the Aorta

**DOI:** 10.3390/diagnostics13243645

**Published:** 2023-12-12

**Authors:** Jan-Lucca Hennes, Henner Huflage, Jan-Peter Grunz, Viktor Hartung, Anne Marie Augustin, Theresa Sophie Patzer, Pauline Pannenbecker, Bernhard Petritsch, Thorsten Alexander Bley, Philipp Gruschwitz

**Affiliations:** Department of Diagnostic and Interventional Radiology, University Hospital Würzburg, 97080 Würzburg, Germany; huflage_h@ukw.de (H.H.); augustin_a@ukw.de (A.M.A.); gruschwitz_p@ukw.de (P.G.)

**Keywords:** CT angiography, aorta, photon-counting-detector CT, radiation dose reduction, spectral imaging

## Abstract

This retrospective study aims to provide an intra-individual comparison of aortic CT angiographies (CTAs) using first-generation photon-counting-detector CT (PCD-CT) and third-generation energy-integrating-detector CT (EID-CT). High-pitch CTAs were performed with both scanners and equal contrast-agent protocols. EID-CT employed automatic tube voltage selection (90/100 kVp) with reference tube current of 434/350 mAs, whereas multi-energy PCD-CT scans were generated with fixed tube voltage (120 kVp), image quality level of 64, and reconstructed as 55 keV monoenergetic images. For image quality assessment, contrast-to-noise ratios (CNRs) were calculated, and subjective evaluation (overall quality, luminal contrast, vessel sharpness, blooming, and beam hardening) was performed independently by three radiologists. Fifty-seven patients (12 women, 45 men) were included with a median interval between examinations of 12.7 months (interquartile range 11.1 months). Using manufacturer-recommended scan protocols resulted in a substantially lower radiation dose in PCD-CT (size-specific dose estimate: 4.88 ± 0.48 versus 6.28 ± 0.50 mGy, *p* < 0.001), while CNR was approximately 50% higher (41.11 ± 8.68 versus 27.05 ± 6.73, *p* < 0.001). Overall image quality and luminal contrast were deemed superior in PCD-CT (*p* < 0.001). Notably, EID-CT allowed for comparable vessel sharpness (*p* = 0.439) and less pronounced blooming and beam hardening (*p* < 0.001). Inter-rater agreement was good to excellent (0.58–0.87). Concluding, aortic PCD-CTAs facilitate increased image quality with significantly lower radiation dose compared to EID-CTAs.

## 1. Introduction

Cardiovascular disease constitutes an increasing concern in an aging population and in patients with risk factors such as hypertension, diabetes mellitus, dyslipidemia, or smoking history [1]. In recent decades, the morbidity and mortality associated with aortic disease have steadily increased [2]. Despite major advances in diagnosis as well as in minimal invasive and surgical treatment, the burden of disease remains high [3]. Computed tomography angiography (CTA) of the aorta plays a central role in the diagnosis, risk evaluation, and follow-up of aortic disease [4], being performed repeatedly in the pre- and post-treatment setting. The resulting radiation exposure is a risk factor for secondary malignancies [5], especially in younger patients with genetic aortic diseases like Marfan or Ehlers–Danlos syndrome [6,7].

Following the “As Low As Reasonably Achievable” (ALARA) principle, reducing the radiation exposure while ensuring diagnostic image quality is crucial, and innovations such as automated tube voltage selection (ATVS) or low-kV imaging have been instrumental for dose saving in energy-integrating-detector CT (EID-CT) [6,8]. With the introduction of dual-energy CT (DE-CT) scanners, an attempt was made to also reduce the amount of contrast medium required by using virtual monoenergetic images (VMI) in order to prevent potential kidney damage. For this purpose, reconstructions with a low keV level close to the k edge of iodine were generated from multienergetic spectral data to maintain the luminal attenuation despite reduced contrast agent dosage. One major disadvantage of DE-CT, however, lies in the higher radiation exposure per scan compared to single-energy CT, especially for abdominal CTA [9,10,11], although a dose benefit is achievable in multi-phase scans due to virtual non-contrast reconstructions. Further limiting DE-CT for aortic CTA, image noise is considerably increased at low keV levels, subsequently hampering diagnostic assessability [12,13].

With the recent introduction of photon-counting-detector CT (PCD-CT) to clinical routine, the CT landscape has been altered substantially. Unlike conventional EID-CT, PCD-CT converts incoming photons directly into electrical voltage, allowing the measurement of each photon’s specific energy. This facilitates spectral imaging without dose penalty, while eliminating electronic noise [14]. Previous studies comparing PCD-CT with conventional EID-CT have shown improved image quality on the one hand and radiation-saving potential on the other [15,16]. However, dedicated intra-individual comparisons of aortic CTA with the two detector technologies are lacking thus far.

Therefore, the aim of this study was to investigate the differences between PCD-CT and EID-CT regarding image quality and radiation dose in patients with consecutive aortic CTAs on both scanners using manufacturer-recommended settings.

## 2. Materials and Methods

### 2.1. Study Design and Ethical Compliance

The local ethics committee granted approval for this single-center retrospective study (protocol number: 20230928 01). The need for additional written informed consent was waived and the principles of the declaration of Helsinki were adhered.

### 2.2. Study Protocol and Patient Population

The institutional database was searched for patients who had undergone PCD-CTA of the thoraco-abdominal aorta between October 2022 and August 2023. Only patients who had previously received a comparable EID-CTA examination with a third-generation dual-source CT scanner (Somatom Force, Siemens Healthineers, Forchheim, Germany) were considered for study inclusion. Additional exclusion criteria were deviations from the standard scan or contrast medium protocols and incomplete scan volumes. A maximum difference of 10 mL contrast medium in favor of EID-CT was tolerated and did not lead to study exclusion.

**Figure 1:**
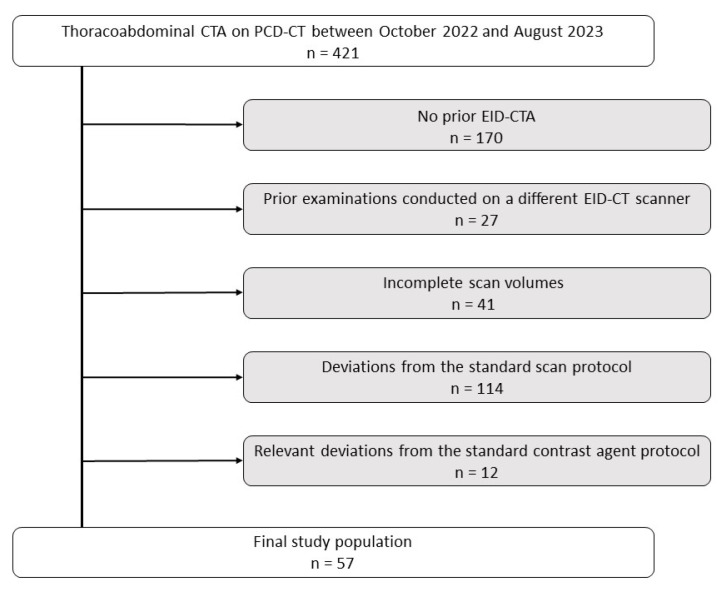
Flowchart visualizing patient exclusion criteria. EID-CT, energy-integrating detector computed tomography; PCD-CT, photon-counting detector computed tomography.

Within the study period, a total of 421 PCD-CTA scans of the aorta were performed at our institution. Of these, 170 examinations were excluded from the study due to the lack of a previous EID-CTA examination. Of the remaining 251 scans, 27 were performed with a different EID-CT scanner, 41 exhibited an incomplete scan volume, 114 displayed deviations from the standard scan protocol, and 12 cases presented relevant deviations from the standard contrast agent protocol. The final study cohort comprised 57 patient examinations (12 woman, 21.1%; and 45 men, 78.9%) with matched pairs of PCD-CTA and EID-CTA examinations (Figure 1). Mean age at the time of imaging was 64.19 ± 10.31 years (PCD-CT) and 62.84 ± 10.13 years (EID-CT). The median interval between examinations was 12.7 months (IQR 11.1 months).

### 2.3. CTA Protocols 

Irrespective of scanner, CTA examinations were conducted with manufacturer-recommended ECG-triggered high-pitch protocols (pitch factor 3.2, rotation time 0.25 s in cranio-caudal direction. PCD-CTA was performed with a first-generation cadmium-telluride-based system (Naeotom Alpha, Siemens Healthineers). Acquiring multienergetic datasets (Quantum plus) with a fixed tube voltage of 120 kVp and a reference image quality index of 64 (dimensionless index to compare image quality between different scan protocols), the standard scan mode with 2 × 2 pixel binning and a collimation of 144 mm × 0.4 mm were used in all patients. EID-CTA were performed in single-energy mode with ATVS (CARE kV, Siemens Healthineers), resulting in scans with either 90 kVp (reference tube current of 434 mAs) or 100 kVp (350 mAs). In EID-CT two detector acquiring modus with 96 mm × 0.6 mm collimation using z flying focal spot was used resulting in an effective collimation of 192 mm × 0.6 mm. Automatic tube current modulation (CARE Dose 4D, Siemens Healthineers) was employed for radiation saving in both cases.

### 2.4. Image Reconstruction

Using a slice thickness/increment of 3.0 mm and a soft reconstruction kernel (Bv 36), an axial image stack was created and analyzed for all CT examinations. Since every PCD-CTA comprises a multi-energy dataset with intrinsic spectral information, monoenergetic images were reconstructed at 55 keV, following manufacturer recommendations. Field of view was kept constant at approximately 350 × 350 mm with slight variations in case of a larger abdominal girth. The pixel matrix for EID-CTA scans was fixed at 512 × 512, while the PCD-CT scanner automatically adjusts the matrix depending on the field of view (512 to 768 pixels). CT protocols and image reconstruction settings are summarized in Table 1.

### 2.5. Contrast Agent Protocol

The standard protocol was specified as follows: 20 mL saline pre-bolus, followed by bolus injection of 60 mL iodine contrast medium (Imeron 350 mg iodine/mL; Bracco, Milan, Italy) and a subsequent 20 mL saline post-bolus. The application was performed via a 20-Gauge peripheral vein catheter and an automatic injector (CT motion, Ulrich GmbH, Ulm, Germany) at a flow rate of 3.5 mL/s. To ensure proper arterial enhancement, bolus tracking was performed with a region of interest (ROI) positioned in the descending thoracic aorta (trigger threshold 100 HU; delay 7 s).

### 2.6. Quantitative Image Comparisons

The intravascular attenuation (*HU_artery_*) was measured using a five-segment model by manually placing ROIs in the ascending thoracic aorta (1), aortic arch (2), descending thoracic aorta (3), infrarenal abdominal aorta (4), and common iliac artery (5). In addition, signal attenuation in muscle tissue (*HU_muscle_*) and the standard deviation of HU values in fat tissue (*SD_fat_*; considered representative of image noise) were measured on the same image slice using ROIs of similar size. Contrast-to-noise ratio without (*CNR*) and with dose-correction (*CNRD*) were calculated using the following formulas:

$$CNR=\frac{\left({HU}_{artery}-{HU}_{muscle}\right)}{{SD}_{fat}}\;\mathrm{and}\;CNRD=\frac{CNR}{\sqrt{{CTDI\;}_{Vol}}}$$

### 2.7. Qualitative Image Comparisons

Image quality was further assessed subjectively using four evaluation criteria: overall image quality, luminal contrast, vascular sharpness, and influence of blooming/beam hardening. Two radiology residents (with 1 and 6 years of training in vascular imaging) and a board-certified radiologist with 9 years of experience in the field evaluated each dataset independently using a five-point rating scale (1: non-diagnostic, 2: poor, 3: moderate, 4: good, 5: excellent), while being blinded to all clinical and protocol-related information.

### 2.8. Statistical Analysis

Statistical analysis was conducted using specialized software (DATAtab Team 2023, DATAtab e.U., Graz, Austria). Metric data were analyzed for normal distribution using Kolmogorov–Smirnov tests. Mean ± standard deviation is reported for continuous variables with normal distribution, while median values (interquartile ranges, IQR) are stated otherwise. To compare objective and subjective image quality parameters, non-parametric *t*-tests or Wilcoxon signed rank tests were employed, with statistical significance being assumed at *p* < 0.050. Of note, for subjective image parameters, the ratings of all three observers were pooled. Kendall’s W was used to determine the level of inter-rater agreement. Coefficients between 0.5 and 0.8 were considered indicative of good agreement, whereas higher coefficients were presumed to indicate excellent agreement [17].

## 3. Results

### 3.1. Patient Characteristics and Radiation Dose

No significant difference in body mass index was found between patients at the time of examination (PCD-CT: 24.68 ± 2.20 kg/m^2^ versus EID-CT: 24.92 ± 2.36 kg/m^2^; *p* = 0.187). Automatic tube current modulation predominantly selected a tube voltage of 100 kVp (52 patients), while 90 kVp was only chosen for five patients. 

**Figure 2:**
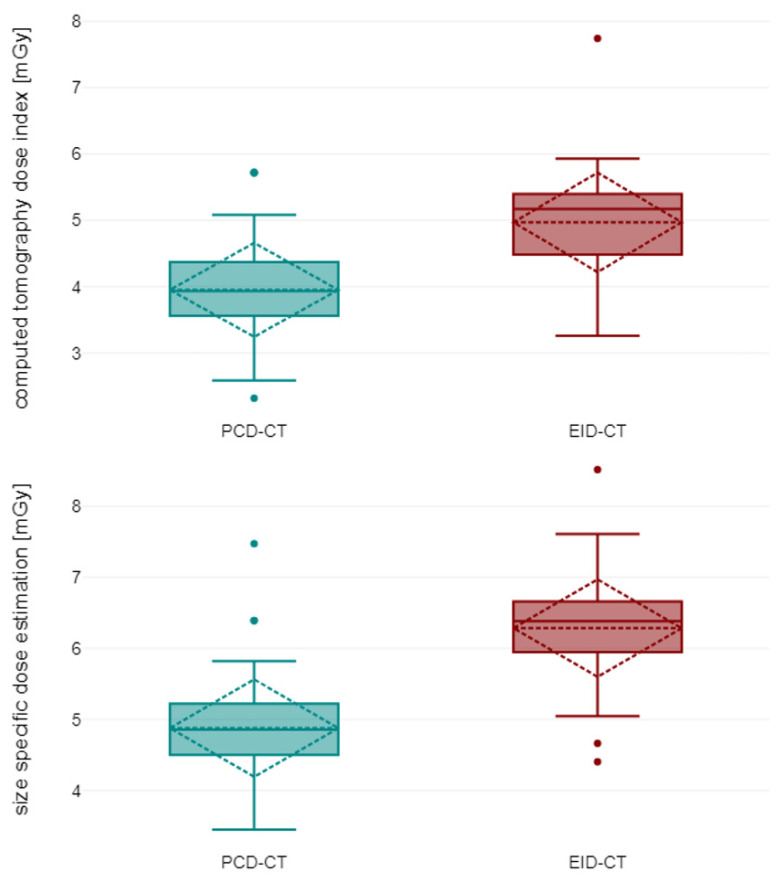
Computed tomography dose index (**top**) and size-specific dose estimation (**bottom**) for photon-counting-detector CT and energy-integrating-detector CT. Boxplots show median and 25%/75% quantile (solid line), minimum and maximum as well as mean value with standard deviation (dashed line). EID-CT, energy-integrating detector computed tomography; PCD-CT, photon-counting detector computed tomography.

Using manufacturer-recommended scan protocols resulted in a significant reduction in volume computed tomography dose index (CTDI_Vol_: PCD-CT: 3.95 ± 0.54 mGy versus EID-CT: 4.97 ± 0.57 mGy; *p* < 0.001) and size-specific dose estimate (PCD-CT: 4.88 ± 0.48 mGy versus EID-CT: 6.28 ± 0.50 mGy; *p* < 0.001) with the novel detector technology (Figure 2). Patient characteristics are summarized in Table 2.

### 3.2. Objective Image Quality

**Figure 3:**
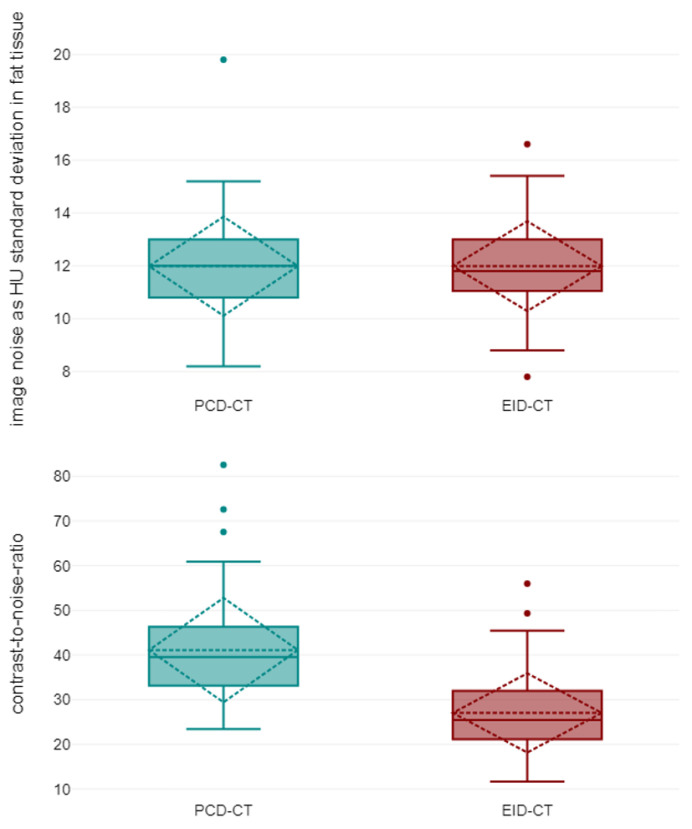
Image noise (**top**) and contrast-to-noise-ratios (**bottom**) in photon-counting-detector computed tomography (PCD-CT) and energy-integrating-detector CT (EID-CT). Boxplots show median and 25%/75% quantile (solid line), minimum and maximum as well as mean value with standard deviation (dashed line).

**Figure 4:**
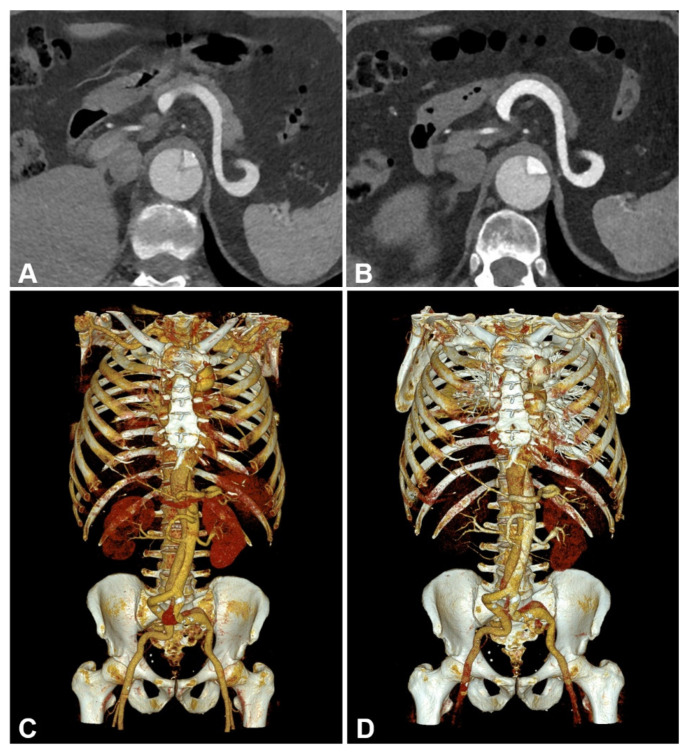
Side-by-side comparison between energy-integrating-detector computed tomography (EID-CT) (**A**,**C**) and photon-counting-detector CT (PCD-CT) angiography (**B**,**D**) in a 61-year-old man with surgically repaired aortic dissection. EID-CT: CTDI_Vol_ 5.93 mGy; SSDE 6.52 mGy. PCD-CT: CTDI_Vol_ 4.58 mGy; SSDE 5.26 mGy. (**A**,**B**) representative axial orientated CTA images (reconstruction kernel Bv36; slice thickness/increment 3 mm; window setting: width 700 HU/center 100 HU) (**C**,**D**) cinematic volume rendering images.

Averaged attenuation in the vascular lumen was significantly higher in PCD-CT (528.53 ± 77.28 HU) than in EID-CT (368.8 ± 62.63 HU; *p* < 0.001), while the used contrast agent dose was equal (60.60 ± 1.30 mL versus 60.69 ± 1.26 mL; *p* = 0.958). Image noise measurements in virtual monoenergetic PCD-CT reconstructions at 55 keV and polychromatic EID-CT scans at 90/100 kVp were equal (PCD-CT: 11.99 ± 1.4 HU versus EID-CT: 11.99 ± 1.32 HU; *p* = 0.943), resulting in an approximately 50% higher CNR when using the PCD-CT (41.11 ± 8.68 versus 27.05 ± 6.73; *p* < 0.001). Taking into account the applied radiation dose, CNRD in PCD-CTA was 70% higher compared to EID-CTA (21.25 ± 5.54 versus 12.33 ± 3.44; *p* < 0.001). Results are summarized in Figure 3 and Table 3. A side-by-side comparison of the achieved CTA images is shown in Figure 4.

### 3.3. Subjective Image Quality

**Figure 5:**
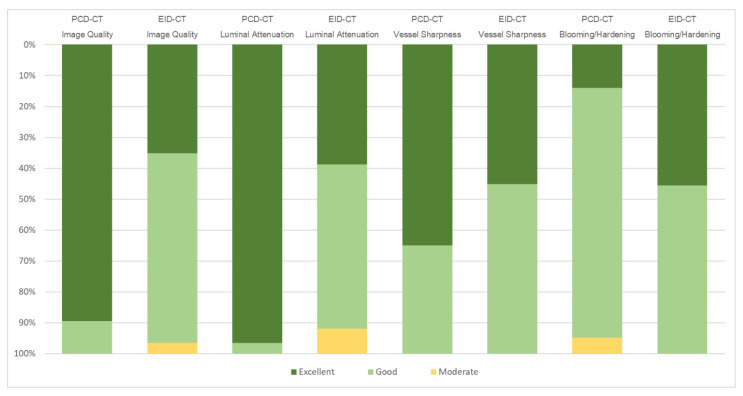
Subjective image quality in photon-counting-detector CT (PCD-CT) and energy-integrating-detector CT (EID-CT).

**Figure 6:**
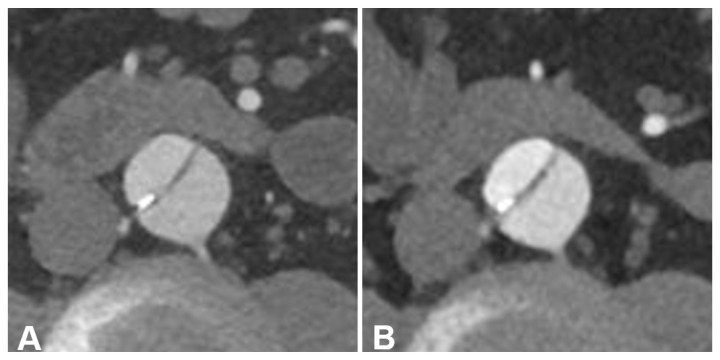
Side-by-side comparison between energy-integrating detector computed tomography (EID-CT) (**A**) and photon-counting detector CT (PCD-CT) angiography (**B**) in a 56-year-old man with TEVAR. EID-CT: CTDI_Vol_ 5.73 mGy; SSDE 6.42 mGy. PCD-CT: CTDI_Vol_ 5.72 mGy; SSDE 6.39 mGy. Representative axial orientated CTA images (reconstruction kernel Bv36; slice thickness/increment 3 mm; window setting: width 700 HU/center 100 HU). (**A**) EID-CT with subjective advantages in terms of blooming/beam hardening (compare wall calcification in the dissection membrane) and vessel sharpness (see dissection membrane). (**B**) PCD-CT with advantages in terms of luminal contrast and overall image quality.

Overall subjective image quality and lumen attenuation were substantially higher for PCD-CT compared to EID-CT (5 [IQR 5-5] versus 4 [IQR 4-5], each; *p* < 0.001). No significant difference in vessel sharpness was ascertained between the two scanners (both 5 [IQR 4-5], *p* = 0.439). Blooming and beam hardening artefacts were considered more pronounced in PCD-CT than EID-CT (4 [IQR 4-4] versus 4 [IQR 4-5]; *p* < 0.001). Inter-reader reliability was good to excellent, with the lowest level of agreement being established for lumen attenuation in EID-CT (W = 0.58) and the highest level of agreement for blooming/beam hardening in EID-CT (W = 0.87). The pooled rating results of all three readers are visualized in Figure 5, while a side-by-side visualization of the differences in the subjectively assessed image characteristics of EID-CT and PCD-CT is shown in Figure 6.

## 4. Discussion

In this retrospective study, we performed intra-individual comparisons of image quality and radiation exposure in aortic photon-counting and energy-integrating detector CT angiographies using manufacturer-recommended scanner settings. The obtained results indicate significant dose reduction potential with simultaneous improvement of image quality when employing the novel detector technology.

Studies dealing with VMI of PCD-CT in vascular imaging recommend different keV levels between 45 and 70 keV for contrasted depiction of the aorta. However, a compromise has to be found between the required intraluminal attenuation and an acceptable image noise level, as these increase progressively at low keV levels [18,19,20,21]. In accordance with the employed PCD-CT system’s standard setting, virtual monoenergetic reconstructions at 55 keV were investigated in the current study. In principle, VMI could also be generated with the used EID-CT scanner. However, since image acquisition in dual-energy scan mode is associated with increased radiation exposure, the clinical use of VMI for aortic assessment is mainly restricted to multiphase examinations, which offset the additional dose burden by using a virtual instead of a true non-contrast phase. Moreover, DE-CT limits the applicable pitch factor, which increases radiation dose and susceptibility to motion artifacts even further [22]. Hence, high-pitch low-kV single-phase aortic imaging has become the standard for EID-CTA [10,11,15,16,23] and is used for comparison to PCD-CTA in the present investigation.

In accordance with previous research, monoenergetic reconstructions of PCD-CT data showed significantly improved intraluminal attenuation over the corresponding low-kV EID-CTA scans. Euler et al. compared different VMI level settings for PCD-CTA with single-energy EID-CTA in a matched patient population [19]. While the authors reported slightly higher vessel attenuation and image noise as well as slightly lower CNR in 55 keV reconstructions, we were able to demonstrate significantly higher CNR based on superior intraluminal attenuation and almost equal image noise. We assume that this discrepancy may be attributed to the overall higher radiation dose applied in our study. With a quasi-linear relationship between radiation dose and CNR in the clinical dose range [24], an additional radiation dose reduction of approximately 35% in PCD-CT would be possible (from about 4 mGy to 2.6 mGy) to achieve the same CNR as in EID-CT. Together with the already 20% lower applied dose of the PCD-CT scan, this results in an extrapolated total dose reduction of about 55%. While the extrapolated CNR of our examinations at the dose used by Euler et al. [19] (CTDI_Vol_ 2.6 mGy) calculated according to the principle mentioned above would be about 15 for the EID-CT and thus comparable with the reported 17 ± 8 by Euler et al., in contrast, the calculated CNR of the PCD-CT scan would be 27 and thus significantly higher than the CNR stated by Euler et al. (16 ± 5). Thus, the differences in image quality shown by our study should remain almost unchanged even if this lower radiation dose would be applied. Further studies are needed to verify this assumption.

In contrast to Euler et al., who ascertained no significant differences between the subjective image quality of 55 keV PCD-CT and conventional EID-CT (subsequently recommending VMI at 40–45 keV as the optimal setting), we were able to demonstrate considerable advantages for PCD-CT VMI at 55 keV regarding overall image quality and intraluminal contrast, while vessel sharpness and influence by blooming or beam hardening were also deemed clinically acceptable. This finding is supported by our objective image quality assessment. It must be mentioned that, irrespective of scanner, no CTA examination was considered diagnostically inacceptable. Therefore, further dose reduction may be feasible with either CT system. Even more aggressive low-dose protocols would particularly benefit younger patients, e.g., with genetic aortic diseases or bicuspid aortic valves, since these individuals require repeated CTA examinations and thus have a high lifetime radiation exposure. Future investigations are certainly warranted to find the minimum tolerable radiation dose for aortic CTA generated with PCD-CT. With that being said, the radiation dose in the present investigation was already low compared to other studies, which applied 3.7 mGy [22] and 8.3 mGy [25], respectively, with the exception being Euler et al., who reported a very low dose of merely 2.6 mGy [19]. However, our results suggest that even this radiation dose could be considerably reduced while maintaining diagnostic quality imaging.

In another recent study, Higashigaito et al. attempted to develop a low-contrast media protocol for PCD-CT with equal radiation dose and image quality [18]. Depending on the CNR gain in PCD-CT, the amount of applied contrast agent was reduced by the same proportion. The authors concluded that a 25% reduction in contrast agent volume (25.9 mg versus 19.4 mg iodine) results in noninferior subjective and objective image quality. However, the contrast agent volume of 60 mL (Imeron 350 mg/mL Iodine) used in our study corresponds to an iodine amount of 21.0 mg, which is only slightly (8%) above the protocol suggested by Higashigaito et al. With this contrast agent protocol, the achievable CNR in EID-CT was about 35% lower than in PCD-CT. Since Higashigaito et al. demonstrated a linear relationship between the administered amount of contrast agent and the resulting CNR, we can assume that applying merely 40 mL of contrast agent (14 mg iodine) in PCD-CTA would be sufficient to match the CNR of EID-CTA with the regular contrast agent volume. By increasing the applied radiation dose up to the national dose reference value (CTDI_Vol_ 7 mGy), i.e., 75% more than used in our study, the required amount of contrast agent could theoretically even be reduced to about 25 mL if PCD-CT is used instead of EID-CT. This finding is especially relevant for patients with renal insufficiency, who could be examined via CTA with a lower risk for acute/chronic kidney failure.

Our results suggest the need for explicit scan protocols adapted to the examined patient population. In our opinion, at least a low-dose protocol to minimize stochastic radiation effects of repetitive CT examinations and a low-contrast protocol to protect the patient’s kidney function should be established. In addition to the dose reduction potential of PCD-CT compared to conventional EID-CT described above, our subjective assessment suggests a diagnostically justifiable image quality reduction below the quality generated by EID-CT by reducing the image quality (IQ) level leading to further radiation dose reduction. A similar situation applies to a low-contrast protocol using a contrast medium quantity of approx. 30 mL (350 mg/mL iodine), optionally with a compensatory elevation of the IQ level with a slight increase in the applied radiation dose, which should lead to diagnostic image quality and contrast-to-noise-ratio. Further studies are required to define the exact scan parameters, eventually using preliminary phantom examinations.

Several methodological limitations have to be acknowledged. First, due to a previous EID-CTA examination being defined as a prerequisite for study inclusion, the investigated sample comprised only 57 patients. Second, the investigated data were collected retrospectively at one tertiary-care university hospital in the form of a single-center study. Third, despite being technically feasible, no ultra-high resolution PCD-CT scans were performed, particularly as the scan time would be more than twice as long compared to standard mode due to the deviating maximum table feed, which is disadvantageous for vascular examinations. Instead, we focused on standard resolution CTA scans to maximize comparability to EID-CT. Fourth, acquisition of multienergetic datasets with a lower kV level (<100 kV) would have been desirable for PCD-CT. However, this setting was not available for clinical use at the time of writing. Fifth, a comparison with VMI of EID-DE-CTA was omitted because dual-energy acquisition has been superseded by low kV single-energy examinations in recent years for this particular imaging task. Sixth, inter-vendor and inter-scanner transferability of results was not investigated. Finally, diagnostic accuracy was not investigated, however, all included examinations were deemed to be of diagnostic quality.

Concluding, aortic PCD-CTA allows for significantly improved subjective and objective image quality while requiring substantially lower radiation dose compared to EID-CTA, when using manufacturer-recommended scan protocols.

## Figures and Tables

**Table 1:** Scan parameters and image reconstruction settings.

Parameters/Settings	EID-CT	PCD-CT
Collimation	192 mm × 0.6 mm *	144 mm × 0.4 mm
Tube voltage	90/100 kVp (ATVS)	120 kVp
Rotation time	0.25 s	0.25 s
Pitch	3.2	3.2
Reconstruction kernel	Bv36	Bv36
Slice thickness/increment	3.0 mm	3.0 mm
Iterative reconstruction level	ADMIRE, level 3	QIR, level 3

ADMIRE, ADvanced Modeled Iterative REconstruction; ATVS, Automatic tube voltage selection; EID-CT, energy-integrating-detector computed tomography; PCD-CT, photon-counting-detector computed tomography; QIR, Quantum Iterative Reconstruction. * two detectors with 96 mm × 0.6 mm fusion z flying focal spot.

**Table 2:** Patient characteristics.

Characteristics	EID-CT	PCD-CT	*p*-Value
Age (years)	62.84 ± 10.13	64.19 ± 10.31	<0.001
Weight (kg)	78.00 (67.00–89.00)	75.00 (65.50–84.50)	0.285
Height (m)	1.78 (1.68–1.88)	1.76 (1.62–1.90)	0.657
BMI (kg/m^2^)	24.92 ± 2.36	24.68 ± 2.20	0.187
CTDI_Vol_ (mGy)	4.97 ± 0.57	3.95 ± 0.54	<0.001
SSDE (mGy)	6.28 ± 0.50	4.88 ± 0.48	<0.001

Normally distributed variables are given as mean ± standard deviation, otherwise we report median values and interquartile ranges in parentheses. BMI, body mass index; CTDI_Vol_, volume computed tomography dose index; EID-CT, energy-integrating-detector computed tomography; PCD-CT, photon-counting-detector computed tomography; SSDE, size-specific dose estimate.

**Table 3:** Image quality parameters. EID-CT, energy-integrating-detector computed tomography; PCD-CT, photon-counting-detector computed tomography; HU, Hounsfield unit.

Image Quality Parameter	EID-CT	PCD-CT	*p*-Value
Vessel attenuation avg. (HU)	368.80 ± 62.63	528.53 ± 77.28	<0.001
Attenuation muscle avg. (HU)	53.80 ± 5.02	47.57 ± 5.80	<0.001
SD attenuation fat avg. (HU)	11.99 ± 1.32	11.99 ± 1.40	0.943
Contrast-to-noise-ratio	27.05 ± 6.73	41.11 ± 8.68	<0.001
Dose-corrected contrast-to-noise-ratio	12.33 ± 3.44	21.25 ± 5.54	<0.001

## Data Availability

The datasets generated and/or analyzed during this study are not publicly available, as CT data and DICOM headers contain patient information. Anonymized data can be obtained upon reasonable request from the corresponding author.
